# Demographic disequilibrium in living nautiloids (*Nautilus* and *Allonautilus*): Canary in the coal mines?

**DOI:** 10.1371/journal.pone.0179811

**Published:** 2017-07-20

**Authors:** W. Bruce Saunders, Emily Greenfest-Allen, Peter D. Ward

**Affiliations:** 1 Department of Geology, Bryn Mawr College, Bryn Mawr, Pennsylvania, United States of America; 2 Institute for Biomedical Informatics, University of Pennsylvania Perelman School of Medicine, Philadelphia, Pennsylvania, United States of America; 3 Department of Biology, University of Washington, Seattle, Washington, United States of America; Virginia Commonwealth University, UNITED STATES

## Abstract

Averaged demographic data from previously unfished populations of *Nautilus* and *Allonautilus* (Cephalopoda) provide a baseline to determine if a population is undisturbed and in “equilibrium” or is in “disequilibrium” as a result of fishery pressure. Data are available for previously undisturbed local nautiloid populations in Papua New Guinea, Australia, Indonesia, Fiji, Palau, American Samoa, New Caledonia and Vanuatu (total *n* = 2,669 live-caught, tagged and released animals). The data show that unfished populations average ~75% males and ~74% mature animals. By contrast, unpublished, anecdotal and historical records since 1900 from the heavily fished central Philippines have shown a persistent decline in trap yields and a change in demographics of *N*. *pompilius*. By 1979, a sample of fished live-caught animals (*n* = 353) comprised only ~28% males and ~27% mature animals. Continued uncontrolled trapping caused collapse of the fishery and the shell industry has moved elsewhere, including Indonesia. In addition, we show that estimated rates of population decline are offered by unpublished tag-release records in unfished Palau. These data show that patterns of trap yields and demographic differences between fished and unfished populations in relative age class and sex ratios can indicate disequilibria wrought by fisheries pressure that can render local populations inviable. Given adequate samples (*n* ≥100 live-caught animals), a threshold of <50% males and mature animals in fished populations should signal the need to initiate curative conservation initiatives. The current trajectory of uncontrolled nautiloid fisheries can only mean trouble and possibly extinction of local populations of this ancient, iconic molluscan lineage.

## Introduction

Arguably one of the most iconic of marine invertebrates, the chambered nautilus includes two genera of nautiloids (*Nautilus* Linnaeus, 1758 and *Allonautilus* Ward and Saunders, 1997), whose pedigrees have roots dating back hundreds of millions of years. While knowledge of the shell may date back to Aristotle [1], the first illustrations of its soft parts trace to Rumpf [2] and to the classic account of Richard Owen [3]. Because of the remoteness of its deep fore-reef habitat in the Indo-Pacific (150-600m), expeditionary efforts were required to study the animal, with most early information gleaned from artisanal fishery efforts and anecdotal accounts [4, 5, 6]. The first modern biological contributions were made in the 1960s [7, 8], and beginning in the 1970s there was a strong surge in nautiloid research that has continued to the present [9, 10].

Recently, it has become apparent that pressure from the shell-trade industry is causing local depletions and fisheries collapses of *Nautilus* populations in the central Philippines and that large-scale trading in shells is now occurring in Indonesia, even though *Nautilus pompilius* is protected there [11, 12, 13]. Since 2008, the U.S. Fish and Wildlife Service along with the National Marine Fisheries Service has been evaluating whether *Allonautilus* and *Nautilus* species should be regulated under the Convention on International Trade in Endangered Species of Wild Fauna and Flora (CITES) [14, 15]. Only recently were sufficient biological information and trade data available to demonstrate the need for regulating international trade of these slow-growing, late-maturing, long-lived marine invertebrates. At the 17th regular meeting of the Conference of the Parties to CITES (CoP17) in 2016 in South Africa [16], a proposal to include the family Nautilidae (Blainville, 1825) in CITES Appendix II submitted by the United States, Fiji, India and Palau was accepted by CITES Parties. Effective January 2, 2017, international trade in all chambered nautilus products must be accompanied by a CITES permit to ensure that such trade is legal and sustainable.

In the Philippines, some fisheries data have emerged regarding historically productive *Nautilus* fishing grounds [11, 12, 16, 17, 18], but the remote, small-scale efforts of artisanal fishermen (often limited to out-rigged dugout canoes with traps pulled by hand) have made obtaining meaningful data difficult. In addition, the fisheries tend to be transient, for once stocks become depleted, shell buyers move on to new areas, with fishermen either following or turning to new resources for income [13, 14, 15, 16]. Additional complications are that there is almost no reporting of nautiloid fishery data and what is available consists of spotty data on totals, with nothing on natural population characteristics such as demographics or trap yields.

Persistent decline in trap yields in the south-central Philippines should have signaled the need for instituting conservation practices such as trapping limits, embargoes, or no-catch refugia. Instead, the fisheries collapsed, and moved elsewhere, primarily to Indonesia [11, 12, 13]. In any case, the current trajectory of nautiloid fisheries can only mean trouble for local populations of this ancient molluscan lineage, but there is currently no empirical basis for evaluating the status of local nautiloid populations that are being subjected to fisheries pressure.

**Fig 1:**
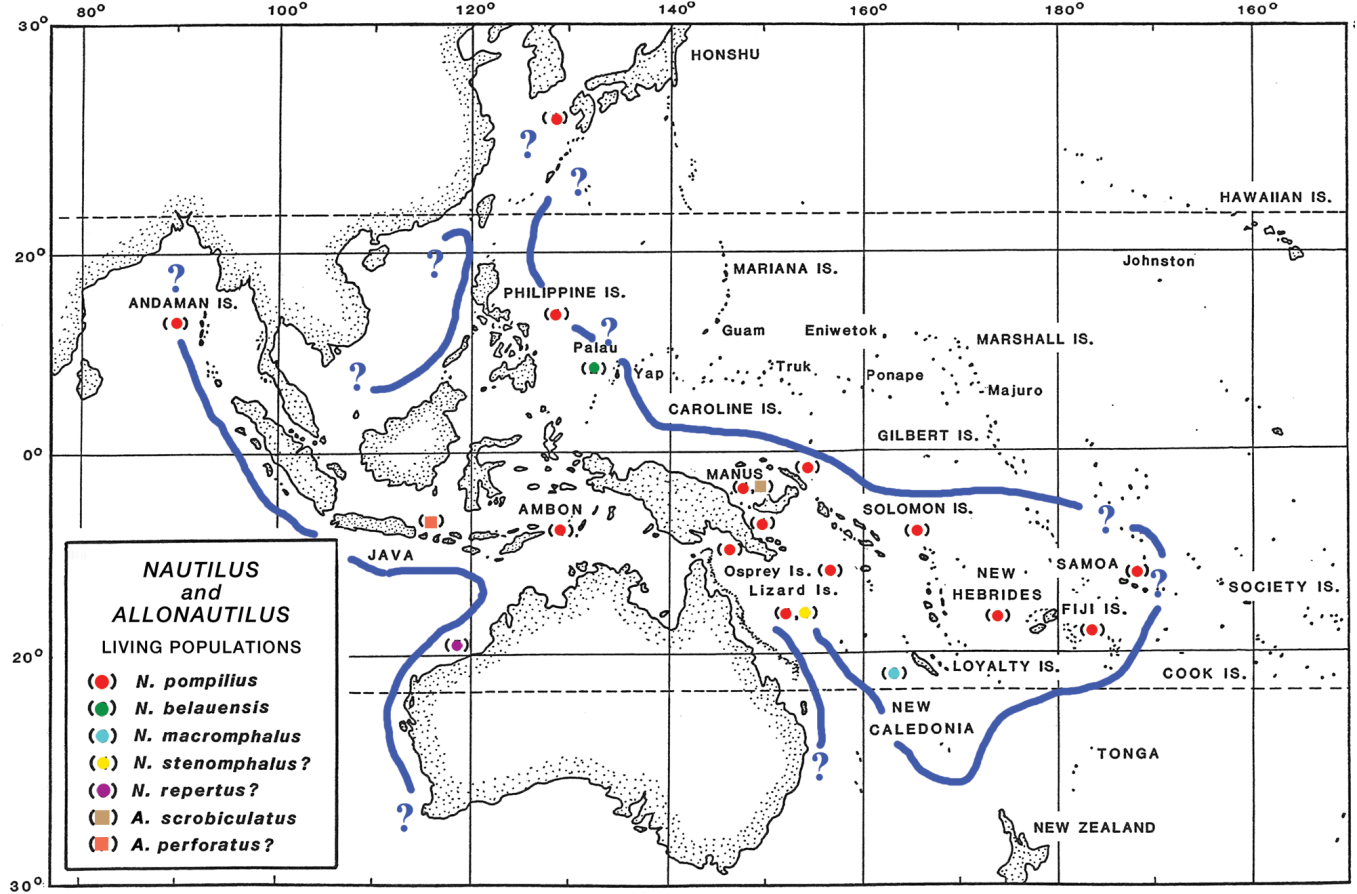
Distribution of living populations of *Nautilus* Linnaeus, 1758 and *Allonautilus* Ward and Saunders, 1997. All plotted populations were sampled for this study except *A*. *perforatus* Conrad, 1849. Nautiloids were not recovered in traps set at Yap, Caroline Is.; Pohnpei and Majuro, Marshall Is.; Kosrae, Gilbert Is.; W. Samoa; and Tonga (WBS). While such negative evidence is not definitive, it is highly suggestive that *Nautilus* does not occur at these sites and aids in establishing distribution limits. Modified from [10] with permission.

**Fig 2:**
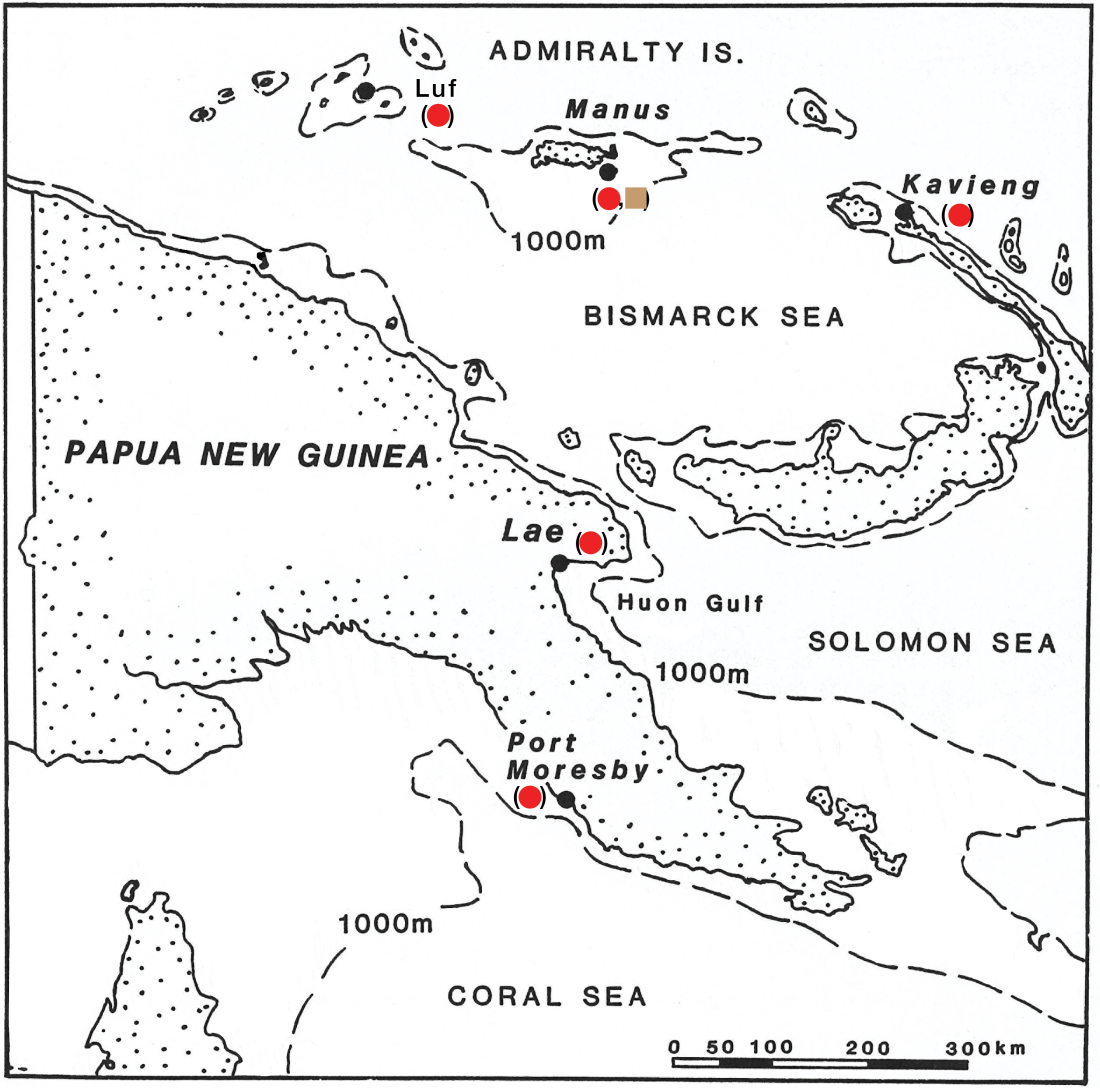
Details of Admiralty Is., PNG, showing trapped nautiloid locations and 1000-meter depth contours. Populations within 1,000 meter contours could have maintained gene flow. At depths >1000 meters, shell implosion would be an isolation factor. Modified from [28] with permission.

Here, we have assimilated demographic parameters from 16 previously unfished populations of *Nautilus* and *Allonautilus* in Papua New Guinea, Australia, Indonesia, Fiji, Palau, American Samoa, New Caledonia and Vanuatu (combined *n* = 2,669 live-caught animals) to characterize undisturbed nautiloid populations in terms of age groupings and sex ratios (Figs 1 and 2; Table A in S1 File). Averaged profiles of these “equilibrium” populations, in turn, are compared to data from the intensely fished Philippines populations that were subjected to uncontrolled exploitation for more than a century. The results show that quantifying the magnitude of demographic disturbance, or “disequilibrium,” provides a basis for evaluating the need for establishing conservation measures to protect this ancient molluscan lineage, particularly given its slow growth, longevity, low fecundity and limiting factors [19, 20, 21, 22, 23, 24, 25, 26, 27].

## Materials and methods

### Taxonomy and genetics of *Nautilus* and *Allonautilus*

Nautiloid genetic studies do not have a long tradition, but there has been a flourish of activity in recent years [29, 30, 31, 32, 33, 34, 35, 36]. By contrast, the taxonomy of extant nautiloids spans more than two centuries, with two genera, eleven species, two subspecies and six variants having been named [10, 36]. The most recent evaluations suggest that five or six species along with two subspecies of *N*. *pompilius* may be valid [10, 36], with the caveat that all but one species was defined from shells alone. Currently, there seems to be agreement that three different phylogenetic clades are recognizable: W. Australia/Indonesia, East Australia/Papua New Guinea, and a West Pacific clade. It is early to attempt integration of taxonomy with these phylogenetic treatments, but a consensus may be developing to treat *N*. *pompilius* as a “superspecies,” with a number of geographically isolated or endemic subspecies: *N*. *p*. *pompilius* Linnaeus,1758 and *N*. *p*. *suluensis* Habe and Okutani, 1988 (which were formally recognized as subspecies [36]); *N*. *stenomphalus* Sowerby, 1849; *N*. *belauensis* Saunders, 1981; and *N*. *repertus* Iredale, 1944. Further progress toward resolution will likely require sampling additional geographically isolated populations.

### Data collection, availability and analysis

The data used here are unmatched for living Nautiloidea. They include the geographic and taxonomic span of all but one named taxon (*A*. *perforatus* Conrad, 1849), and all specimens were live-caught. Most of the data are reported here for the first time and were obtained in conjunction with a survey of the geographic distribution of *Nautilus* for genetic, ecologic, and morphologic studies made during the late 1970s thru 1980s (WBS, PDW). At that time, a few populations had been the subjects of earlier studies (New Caledonia, Fiji, Palau), but only the Philippines had been exposed to long-term uncontrolled fishery pressure.

The dataset was extracted primarily from original field data logs recorded from 1978 to 1987 [37]. Demographic data from Osprey Reef are from published accounts by A. Dunstan *et al*., summarizing observations and field measurements made from 1998 to 2008 [11, 12]. Most of the populations were previously unknown and undisturbed and are used in a demographic assessment context for the first time (with the exception of Osprey Reef, Australia [11, 12]). The majority of animals (~90%) were tagged and released.

Individuals from each population were documented with details on shell morphometrics, weight, sex, relative maturity, trapping data (location, depth, soak time, bait), and additional notes on epizoans, octopod predation, injuries, etc. Baited deep-water remote cameras were deployed at some sites [20].

*Nautilus* trapping involves many variables and vagaries, including trap design, depth, bottom topography, currents, soak time, bait and luck. The trapping efforts reported here were designed to minimize such variables; any vagueness in reporting such details (such as trap design and soak time) reflect an effort to minimize exploitation of these animals, which, as shown here, are clearly vulnerable to over-harvesting.

Demographic data include sex and relative maturity of each animal, following established protocols [37, 26]. Sex determination is straightforward in mature animals (*e*.*g*., presence of nidamental glands, lack of a spadix, the shell of females is smaller and the aperture is noticeably compressed [26]). Characteristics of mature animals (maximum growth achieved) include a sinuous aperture (ocular and hyponomic sinuses), thickened shell margin, and a blackened apertural margin in many individuals (but see comments on Osprey Reef data below). The data logs record maturity categories as follows: mature or barely mature (M, MB) and immature or barely immature (I, IBM). A separate juvenile category (<50% mean shell diameter) was observed, but is not included here because they are so rare (<0.01%) that their differentiation would have negligible effect on demographic analysis.

All data underlying text figures are available in the supporting information (S1 File). These data were all compiled from field notebooks recorded by WB Saunders: “Zoological and morphometric data based on material collected from Palau (Belau), Philippine Is., Papua New Guinea, and elsewhere, during the years 1977–1987 (Part I, 110pp; Part II, 87pp.) 2017.” [37]. The notebooks are in the Invertebrate Zoology Collections, Smithsonian National Museum of Natural History, Washington DC. To request access and copies, contact the Collections Manager (http://invertebrates.si.edu/1IZcontact.htm).

Data analysis involved calculation of the individual and aggregated means of 16 previously unfished populations of *Nautilus* and *Allonatilus* for comparison to the heavily fished Philippine populations from published historical accounts (1900–2015) and to detailed demographic data assembled in 1979 during a research cruise of the NOAA *RV Alpha Helix* (WBS; Table B in S1 File). Trap yields of newly caught and recaught animals in Palau, 1982, were plotted against elapsed time and correlation and *p*-values calculated using R, version 3.3.3 [38].

### Research permits

During the 1970s and 1980s, formal research permits for each country were rarely required and permission from departments of Marine Resources or Fisheries at the national or local levels sufficed for visiting investigators. This is true for American Samoa, Western Samoa, Fiji, Papua New Guinea, Tonga, and Vanuatu. Sites where formal permitting procedures were followed include Palau (Micronesian Mariculture Demonstration Center, Marine Resources); Indonesia (Prof. Dr. Emil Salim, Minister of Environment; Dr. Aprilani Soegiarto, Deputy Chairman for Natural Sciences [LIPI], through Operation Raleigh [U.K.] with support of Dept. of Fisheries, Univ. Pattimura, Ambon); Philippines (1979 *RV Alpha Helix* Comparative Cephalopod Cruise to Tañon Straits [University National Oceanographic Laboratory Systems, Scripps Institution of Oceanography] and Prof. Angel Alcala, Silliman Univ.); Australia (Great Barrier Reef Marine Park Authority and GBR Lizard Is. Research Station and Western Australia Museum, Perth); and New Caledonia (Institut Français de Recherche Scientifique Pour le Développement en Coopération [ORSTOM], Centre de Nouméa).

## Results

**Fig 3:**
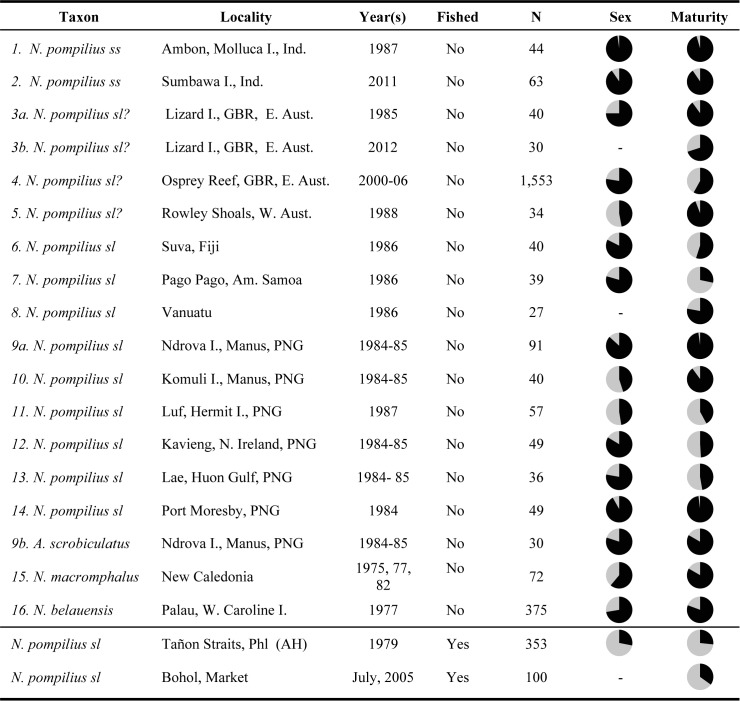
Comparison of sixteen unfished, equilibrium nautiloid populations to a fished, disequilibrium Philippines population reveals significantly higher proportions of males and mature individuals in the equilibrium populations. Demographic data from 16 unfished populations of *Nautilus* and *Allonautilus* (2,669 live-caught, tag-release animals) averaged 75% males and 74% mature animals compared to a 1979 sample of overfished *N*. *pompilius* (*n* = 353) from Tañon Strait, Philippines (~28% males and ~27% mature animals). The Philippine fishery crashed in the 1980s. Pie charts show proportion of males and mature animals in black. For additional details see Table A in S1 File.

This study represents the first demographic summary of a series of isolated, unfished nautiloid populations undertaken to establish averaged or equilibrium nautiloid population profiles. The demographics of these unfished populations are compared to that of the best-known *Nautilus pompilius* fishery, located in the south-central Philippines, which was subjected to uncontrolled fishing for over a century. The comparison reveals striking differences in terms of the percentage of mature animals and sex ratios (Fig 3), which can be used to identify whether local populations are in fishery-induced disequilibrium and should be protected.

Most of the populations were originally targeted for genetic and morphologic analysis; consequently, sample sizes tend to be small and range from *n* = 27 to *n* = 275 individuals (mean *n* = 65). In some cases, smaller sample numbers (*n* < 40), likely resulted in anomalous demographics. For example, when the Kavieng, PNG sample was increased from *n =* 49 to 99 (PDW, Aug., 2015), the total percentage of mature animals increased from 24% to 74% and the percentage of males from 49% to 91%, both of which are much closer to the aggregate population mean. Thus, for future estimates of fisheries impact on nautiloid demographics, we recommend a population sample of at least 100 animals.

### Historical summary of *Nautilus* demographics in the best-known fished and unfished populations

For comparative purposes, a historical summary of the decline in the single best-known *Nautilus pompilius* fishery (the south-central Philippines) is presented here, followed by summaries of the three best-known unfished populations (Osprey Reef, Australia; Palau; and New Caledonia).

### South-central Philippines: History of an uncontrolled *Nautilus* fishery

There has been fishery pressure on *Nautilus pompilius* in the south-central Philippines for more than a century. In 1900, L. E. Griffin [4] described typical trapping practices off Negros Island:

“… baited with meat, in six or eight hundred feet of water. …. Every morning these traps are drawn up for inspection, and a single one sometimes contains four or five live Pearly Nautilii; which are sold for food, bringing about 4 cents apiece.” (p. 104).

In 1901, Bashford Dean [5] provided a similar account of practices and yields in the Tañon Strait, between Cebu and Negros, observing that the trap “… is allowed to remain for several days, often a week or longer” and that “… as many as twenty shells having been taken in a single basket.” (pp. 22, 23).

Seventy years later, Norine Haven [17] reported that, for the most part, *N*. *pompilius* was an incidental by-catch in fish traps. She did locate four fishermen exclusively trapping for *Nautilus*, using traps similar to those described by Dean [5], with overnight catches of 0 to 19 animals, and averaging 5 animals [17].

In 1979, the U.S. National Science Foundation sponsored two cephalopod research cruises (RV *Alpha Helix*) to the Tañon Strait, but few fishermen were still engaged in the fishery. At that time, M. Vailoces (who had earlier assisted Haven) reported that trap yields were so low (~two per trap) that there was little trapping activity (WBS). *Nautilus* purchases were quite limited until rewards were offered (US $5 per live animal, without preference to maturity or sex). This re-activated the fishery for the duration of the *Alpha Helix* cruise, and detailed records of 353 live *N*. *pompilius* purchased from fishermen were obtained [37] (see Table A in S1 File). Only 26.6% were mature and 28% were males. In a random sample of 100 shells for sale in the Bohol Market (2012), only 35% were mature (PDW).

**Fig 4:**
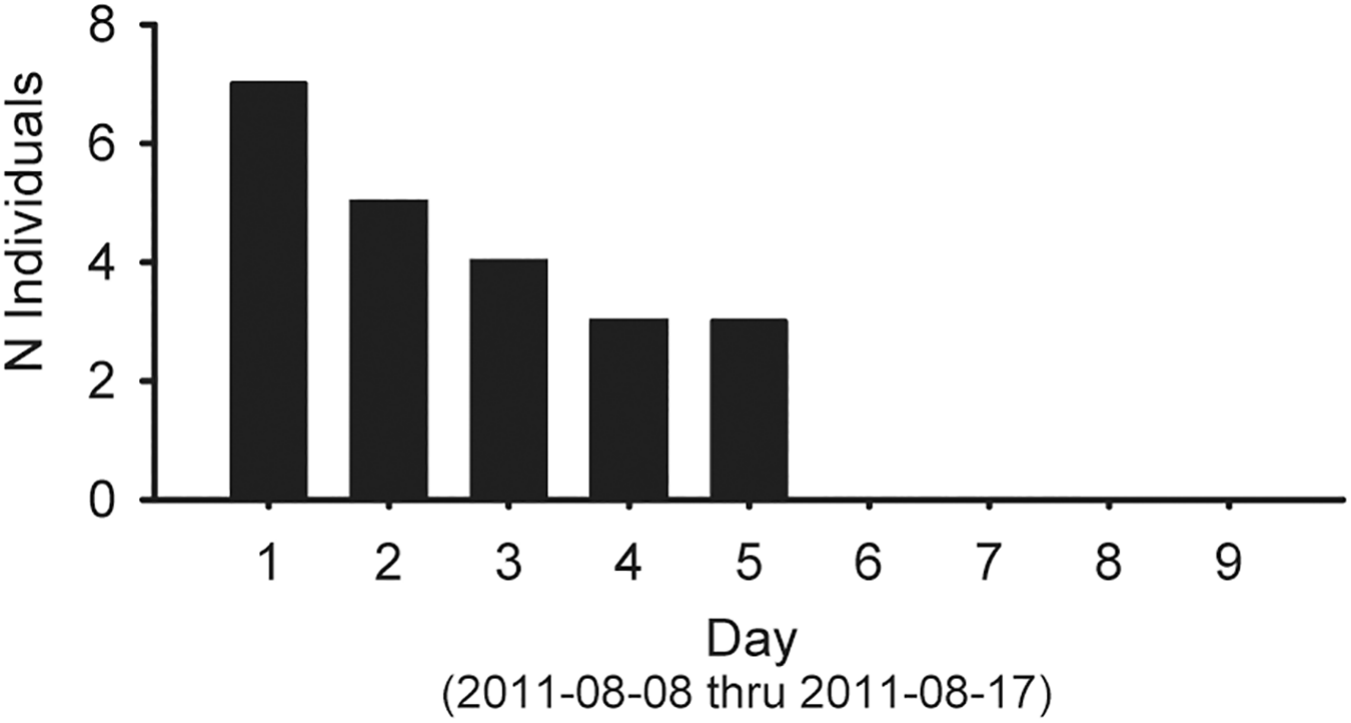
Rapid local decline in N. pompilius trap yields reflects crash in Philippines fishery. Data from three strings of ten traps set overnight at 250-300m depth off Panglao Island in 2005 by P Ward and G Barord (2011) show that Nautilus yields rapidly declined and were not replenished. All animals were marked and released at a site ~10km away and one animal was recaught at the original site.

Baited deep-water cameras set during the 1979 cruise (WBS) recorded a maximum of only one or two *Nautilus* in a ~2m field of view spanning **~**18hr overnight intervals [24]. Identical camera sets in Palau the same year averaged ~9 animals [20]. In 2014, similar low levels of activity were obtained in baited remote underwater video stations (BRUVS) footage in the same region [39], indicating that population density had not returned to pre-1970 levels, even though *Nautilus* fishery efforts in the area had essentially ceased. Since the demise of the central Philippines fisheries, efforts have shifted elsewhere, but trap yields there have been reported as being greatly depleted (by **~**80% over 17 years for Palawan fisheries [11]). In 2011, overnight yields for 10-trap strings set for three consecutive nights plummeted from a total of seven to zero (Fig 4). In summary, it is hard to dispute the historical decline in *Nautilus* in the Philippines over the last century.

### Palau (Belau): An unfished *Nautilus* population

The first Palauan *Nautilus* was caught around 1975 by photographer D. Faulkner using traditional traps [40]. During 1977–1982, several thousand animals were trapped, tagged and released, providing an unparalleled data set [37]. *Nautilus* research in Palau largely ceased in 1982, but *Nautilus* was known to occur there and was publicized as “eco-friendly” for photo-op tours, which are still being promoted by Palau dive operators. Neither commercial trapping nor evidence of population depletion has been documented [41]. Currently *N*. *belauensis* is protected and a permit is required for research (pers. commun., B Carlson, 2016).

Of 375 animals trapped in 1977, 80.5% were mature and 72.3% were male (Fig 3; Table A in S1 File). Data on thousands of additional Palauan *Nautilus* were recorded subsequently and the figures are similar [37], but the 1977 data are used here because they are representative and the (hereto unpublished) population demographics were compiled from field notes [37] along with morphometric data for a published study of sexual dimorphism in *Nautilus* [26].

### Osprey Reef, GBR: An unfished *Nautilus* population

Osprey Reef, GBR was the site of long-term studies (1998–2008), with the most detailed data available for 2000–2006, when 1,553 animals were trapped and released [11, 12]. 82.7% of animals judged mature enough to determine sex (>115mm in diameter) were male. Australian *Nautilus* are protected and permits are required for *Nautilus* research. The Osprey Reef data differ as follows:

1. Osprey Reef *Nautilus* were defined as mature by the presence of a blackened apertural margin [11, 12]. In other populations, in addition to a blackened aperture, maturity has also been defined by the presence of a thickened, sinuous terminal aperture and ocular or hyponomic sinuses [26, 42]. This difference may in part explain the lower proportion of mature animals observed in Osprey Reef (58%) compared to other populations [11, 12].
2. Trapping at Osprey Reef involved 12h (dusk-dawn) soak times (mean yield 6.4 *Nautilus* per trap [11, 12], compared to three or more nights soak time elsewhere. The differences are not critical, as only general comparisons of trap yields are made here.

### New Caledonia and Loyalty Islands: Fishery status uncertain

Until the 1970s, the New Caledonia region had almost no trapping for the endemic species *N*. *macromphalus*, although a number of research studies were based there [8, 9]. However, R. Grandperrin and C. Duflo (Centre ORSTOM, Nouméa, N.C.) documented extensive commercial trapping, spanning 1976–1989. The manuscript (to our knowledge never published) focuses on fisheries aspects based on >3,000 traps and a total catch of >7,000 *N*. *macromphalus* [43]. It would be most helpful for conservation purposes to see this study published and to conduct follow-up trapping to see whether this population is in equilibrium status; given the time lapse since trapping (28–41 years), the population is assumed to have returned to equilibrium.

In 2012 there was no fishery for *Nautilus* and no interest in the animal commercially (EGA). *Nautilus* shells for sale locally were *N*. *pompilius*, probably imported from the Philippines. There was a perception that *Nautilus* was protected. Locals reported fishing for *Nautilus* with hand lines for food while spear fishing, but trapping was unknown.

Demographics presented here for *N*. *macromphalus* are considered representative of an unfished population because at the time they were obtained (PDW, 1975–1982) only limited trapping efforts were being undertaken by a few researchers. A complete set of data is available for 72 specimens [37]; the sex ratio is 61.1% male and 83.3% were mature (Fig 3; Table A in S1 File).

### Combined demographic summaries for unfished and fished nautiloid populations

**Fig 5:**
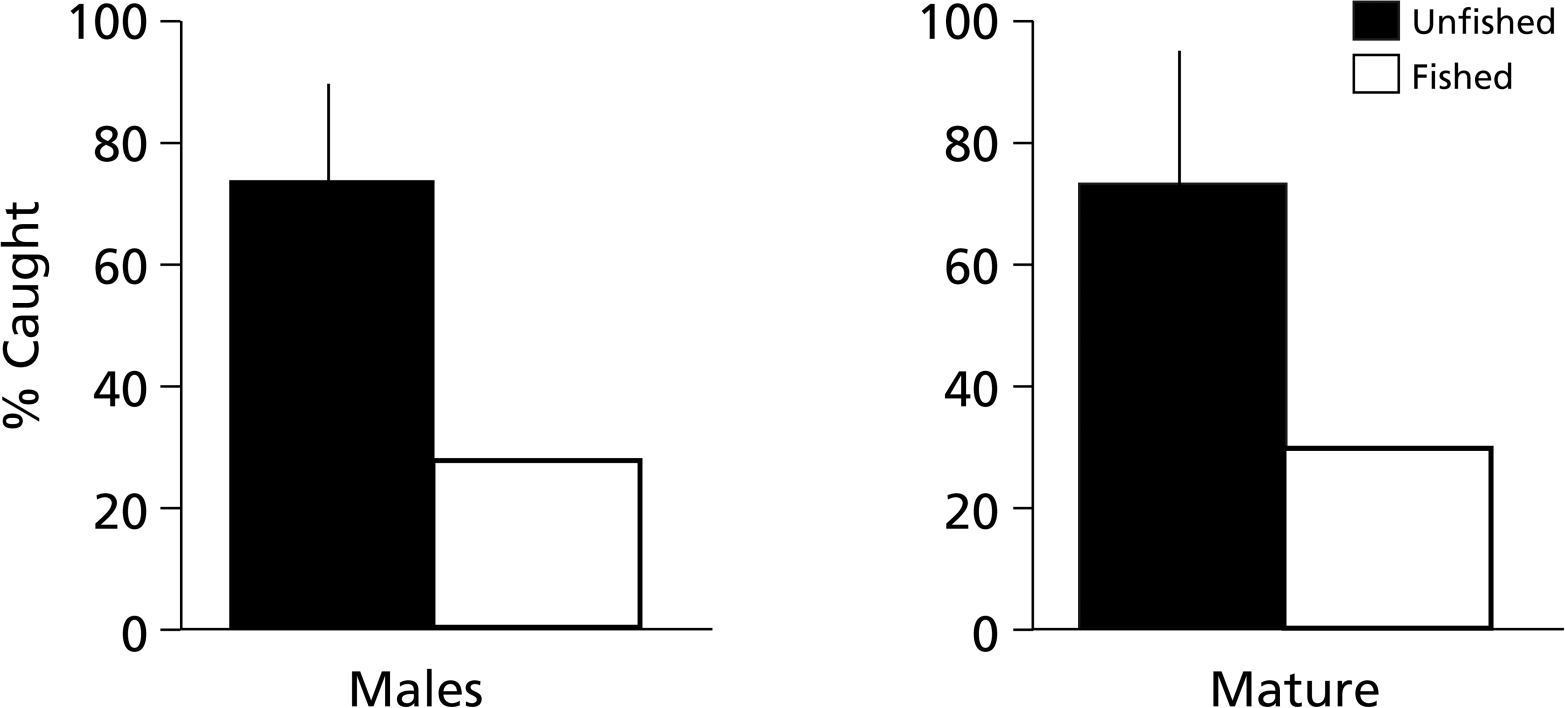
Contrast between unfished (equilibrium) and fished (disequilibrium) nautiloid populations based on live-caught animals. Comparison of the proportion of males and immature individuals in 16 unfished, populations (black, *n* = 2,669; 1975–2012) to a heavily fished population (white; *n* = 353; 1979; Tañon Strait, Philippines) emphasizes the marked relative immaturity of and lack of mature males in the fished population, suggesting a state of disequilibrium. For additional details see Table A in S1 File.

The *combined* total unfished nautiloid sample (*n* = 2,669) includes 1,768 (66.2%) mature nautiloids (males and females). But, as noted above, this reflects a different definition of maturity in the Osprey Reef sample. Therefore, the averaged mean of matures in each *individual*, unfished population (73.9%) is used here as the mean proportion of mature animals. The combined total sample of sexed nautiloids (*n* = 2,488) includes 1,990 males (75%) in unfished populations. We propose that these proportions (~75% males and ~74% mature animals) are equilibrium figures that can be used as baseline approximations for undisturbed local nautiloid populations, ideally based on samples of at least 100 sexed animals and using agreed upon criteria for judging maturity (Fig 5; Table A in S1 File).

By contrast, the 1979 *Alpha Helix* samples from the south-central Philippines included 259 immature individuals (73.4%; Fig 3; Table A in S1 File). At the time, trap yields were so low (1–2 *N*. *pompilius* per trap) that trapping had essentially ceased. Clearly, by 1979, *Nautilus* population in the historic Tañon Straits region was in serious disequilibrium. It was subsequently reported [11,12, 39] that the fisheries had locally collapsed and moved elsewhere, after more than a century.

It is not clear whether the early history of trapping in the Tañon Straits had been sustainable or how rapidly the demise of local *Nautilus* fisheries took to reach unsustainable levels. Such factors as lack of awareness that there might be a problem, lack of data, and introduction of more modern fishing techniques and technologies all played a role.

### How long to reach disequilibrium? Projected rate of fisheries collapse in an unfished population (Palau)

Details of trap yields and tag-release records from Palau provide insight into possible depletion rates of natural *Nautilus* stocks using updated techniques and unlimited trapping. In 1982, an intensive tag-release effort was undertaken (WBS) in order to secure wild growth-rate data and to use remote deep-water cameras to study *Nautilus* [19, 20]. Trapping was undertaken at two localities on the outer fringe reef on opposite sides of Palau (Ngemelis I. and Mutremdiu Point) [26]. 38 traps were set at depths ranging from 150 to 300m; the elapsed trapping period was 87 days. A total of 901 animals were caught for the first time and released; the mean of the first-time caught animals per trap was 24 animals. In addition, 163 (15. 3%) were recaught and released again (Table B in S1 File). Mean trap yield was 28 animals per trap (max. *n =* 66), with soak times targeting 3 nights, but ranging from 1–14 nights (due to inclement weather).

**Fig 6:**
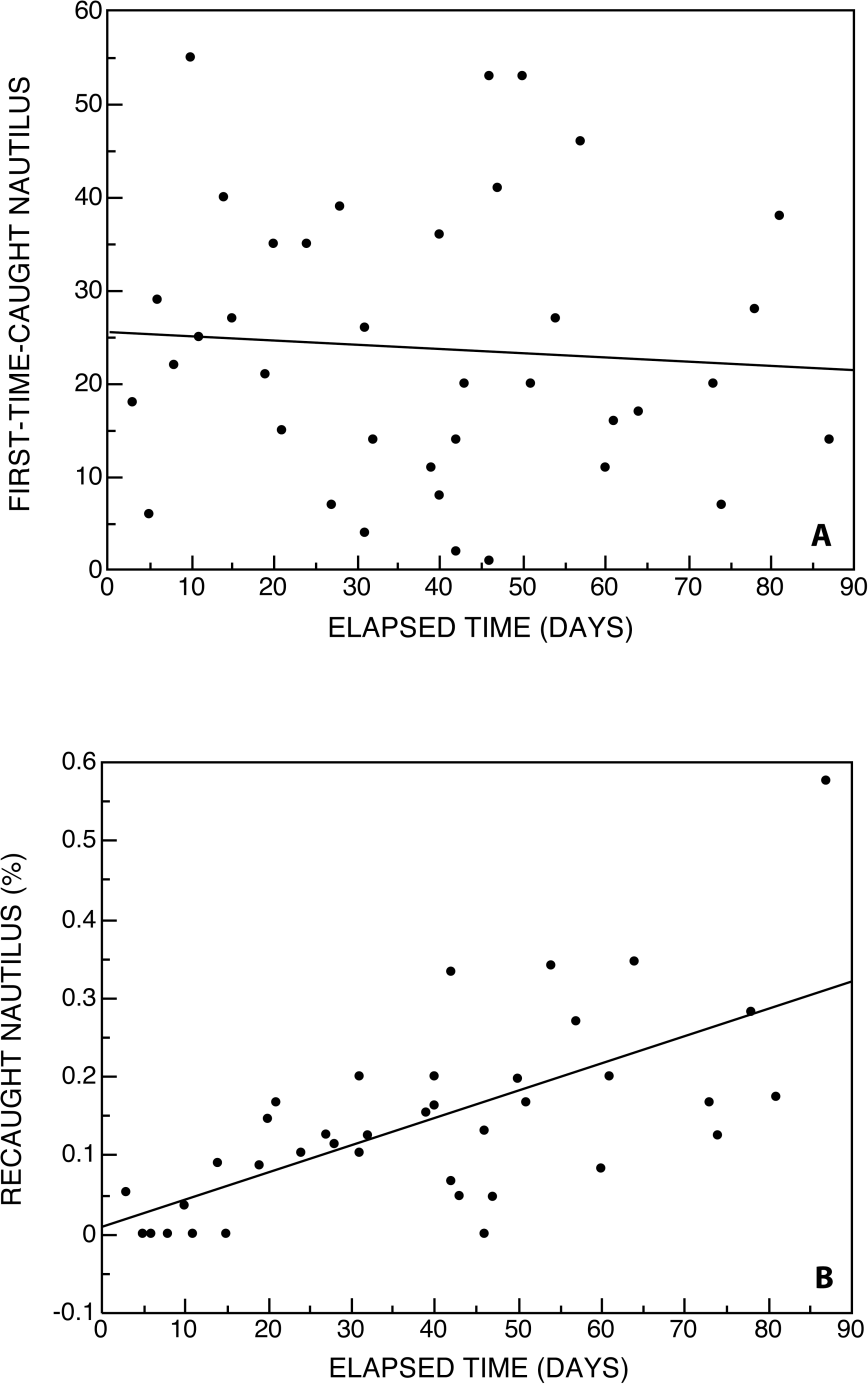
First-time caught and recaught *Nautilus belauensis*, Palau, 1982, suggest trap habituation. **A.** The total number of first-time-caught *Nautilus* per trap varied widely and was uncorrelated with elapsed time (*r*^2^ = -0.07; *p*-value = 0.6722). **B.** By contrast, there was a strong positive correlation between the proportion of recaught *Nautilus* per trap and elapsed time (*r*^*2*^ = 0.579; *p*-value = 0.0001395). Recatch rates >25% were common by the end of the trapping period and some animals were recaught as many as six times, suggesting they were habituating to trap sites as feeding stations and were learning bait access.

The 1982 Palau tag-release data suggest that, although first-time *Nautilus* catch rates fluctuated widely, repeated trapping and release at the same sites seemed to habituate animals so that they more efficiently located and gained entrance or re-entrance to the baited traps (Fig 6). This is supported by remote photosequence records at Mutremdiu spanning ~18hr periods showing that *Nautilus* stays at baited sites for extended periods, and may exit and re-enter baited traps repeatedly [20].

Perhaps most important to fisheries and conservation considerations is the increase in the number of tagged animals recaught at baited trap sites over time. Overall, 15.3% animals were recaught, and the proportion climbed to as much as 58% at the end of the trapping period (Fig 6B). These patterns clearly indicate high susceptibility of the animal to repeated trapping and to habituation of trap sites as feeding stations. If permitted, fishing strategies exploiting this pattern would rapidly deplete local stocks, probably within a matter of months, at least temporarily.

It should be noted that this level of vulnerability to overfishing may be unique to smaller, depth-isolated and endemic populations like Palau, where replenishment from adjacent populations is not readily achievable because of isolation barriers such as geography, water temperature and implosion depth >800m [24, 28]. Such barriers do not exist parallel to the margins of larger land masses (*e*.*g*., Papua New Guinea, Indonesia, Philippines), where movement within optimal depth range (<800m) and natural restocking could occur over thousands of km parallel to coastal areas (Fig 2).

### Disequilibrium in nautiloid populations: Canary in the Coal Mines?

As shown here, comparison of multiple undisturbed *Nautilus* and *Allonautilus* populations to heavily fished and depleted *N*. *pompilius* populations in the south-central Philippines (Figs 3 and 5) shows striking contrasts, as follows:

1. Trapping results for undisturbed *Nautilus* and *Allonautilus* in equilibrium average ~74% mature animals and ~75% males. These averaged unfished demographic data can be used to determine whether disequilibrium wrought by intense fisheries pressure exists. We recommend a sample size of at least 100 individuals and estimate that “alarm” values are reached when mature animals and males in the population sample fall to <50% of mean equilibrium values. Thus, 1979 levels in the central Philippines (~28% males and 27% matures) should have signaled pending collapse of local fisheries, indicating that immediate conservation action was warranted in order to prevent the loss of local population viability that subsequently occurred there.
2. Reduced trap yields are an obvious indication of fisheries pressure (*e*.*g*., 1979 Tañon Straits yields fell to <1–2 per trap). However, using trap yields to estimate population equilibrium will require diligence in monitoring yields as well as such variables as depth, technique, seasonal variability, etc., and this may be challenging given the typically small-scale nature of deep-water trapping for *Nautilus*.
3. The 1981–1982 trapping protocols in previously unfished Palau are not directly comparable, but traps there were ~14 times more productive (mean 28 per trap) than in the central Philippines (which had declined to 1–2 per trap in the 1970s). In Palau, recapture rate averaged ~15%, increasing to ~20–30% within 90 days. Projections indicate that at this rate, if uncontrolled, local fishery crashes in Palau (*e*.*g*., Mutremdiu and Ngemelis) would probably occur within 1 year. Furthermore, because of Palau’s isolation by depth and distance from adjacent populations, stock replenishment would be unlikely to occur quickly, if at all.

In sum, *Nautilus* population disequilibrium will result from uncontrolled overfishing and can be recognized from trapping demographics by anomalously low proportions of males and mature animals. Furthermore, modern equipment and techniques (as utilized in Palau, 1982) might cause trap yields to increase temporarily, only to quickly fall so low that local fisheries are no longer sustainable and that viable populations are threatened. In the central Philippines, the current fisheries situation may have taken as long as a century to reach unsustainable levels. But this slow rate of decline may be due to the small scale of the operations as well as to the traditional technology employed. Exporting mechanized technology to new, nearby areas (*e*.*g*., Bohol, Panay, Panglao) or to more distant, unfished regions (*e*.*g*., Indonesia, Papua New Guinea, Palau, American Samoa) may serve to temporarily vitalize fisheries and shell trade, but it will be at the cost of more rapid disturbance of normal population equilibria, if the 1982 Palau trapping record is any indication.

Conservation measures could include imposing trapping limits based on sex or maturity, regulated trapping seasons, no-fish refugia, or broader alternatives such as import/export quotas or CITES listing as threatened or endangered taxa. In any case, it seems clear that the current, largely uncontrolled exploitation trajectories can only mean trouble for this ancient molluscan lineage.

## Supporting information

S1 File Table A. **Demographic data from 20 living populations of fished and unfished *Nautilus* and *Allonautilus*.** Total *n* = 3,122 sampled animals. Osprey Reef, Aust., data are from Dunstan *et al*. [5, 6]. A catalog of all data except Osprey Reef and Bohol Market is reposited with the USNM Smithsonian Institution, Washington, DC [1]. For an explanation of maturity and sex determinations see [7, 8]. Two localities are listed as separate populations: (1) Lizard Is, GBR, Australia was sampled separately in 1985 (13a) and 2012 (13b); (2) at Ndrova Is., Manus, PNG, both *Allonautilus scrobiculatus* (9a) and *Nautilus pompilius* (9b) occur sympatrically. *N*. *stenomphalus*? is a questionable species found only at Lizard Is., GBR [4], that is included here as *N*. *pompilius sl?* For additional information, see Table A—Notes. **Table B. Trapping results from Palau, 1982, for *Nautilus belauensis* Saunders, 1981.** Traps were set at two sites on the outer fringe reef on opposite sides of Palau (Ngemelis I. and Mutremdiu Point Short Drop Off). Identical box traps were set 38 times at depths of ~150 to 300 meters over a span of 87 days. A total of 901 animals was caught for the first time (“new”) and released and 163 (15. 3%) were recaught and released again. Mean trap yield was 28 animals per trap (max. *n =* 66), with soak times of 1–14 nights, and the mean was 24 new animals per trap (Fig 6). Data compiled from [1]; see Table A—Notes.
(XLSX)
